# Supplementary material for: Splice-Junction-Based Mapping of Alternative Isoforms in the Human Proteome
Source: Cell Rep. Author manuscript; Available in PMC 2020 Jan 15. (PMC6961840; doi:10.1016/j.celrep.2019.11.026)
Supplement: 2 [file NIHMS1546469-supplement-2.zip › DF1/PXD006675/CardiacFibroblast/CardiacFibroblast_4_NEXN_AEQVNEDEENQDTAK.pdf]

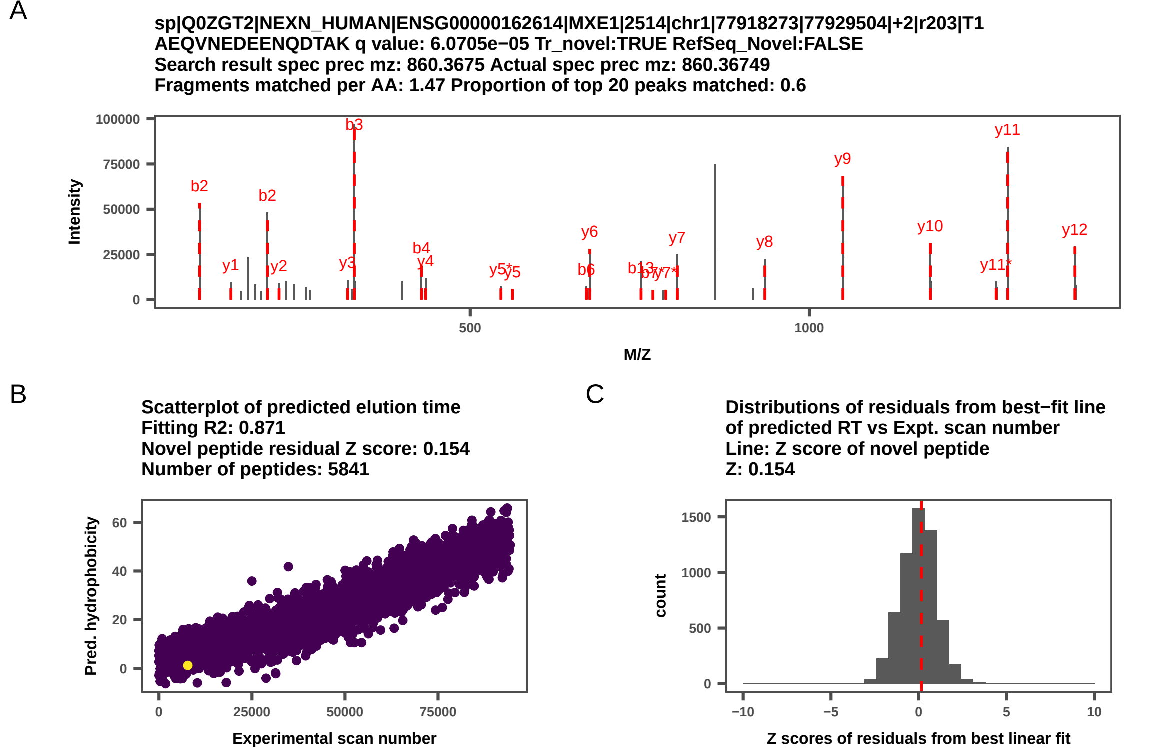
A
sp|Q0ZGT2|NEXN_HUMAN|ENSG00000162614|MXE1|2514|chr1|77918273|77929504|+2|r203|T1
AEQVNEDEENQDTAK q value: 6.0705e−05 Tr_novel:TRUE RefSeq_Novel:FALSE
Search result spec prec mz: 860.3675 Actual spec prec mz: 860.36749
Fragments matched per AA: 1.47 Proportion of top 20 peaks matched: 0.6
Intensity
100000
75000
50000
25000
0
b2
y1
b2
y2
y3
b3
b4
y4
y5*
y5
y6
b6
b13
b7
y7*
y7
y8
y9
y10
y11*
y11
y12
500
1000
M/Z
B
Scatterplot of predicted elution time
Fitting R2: 0.871
Novel peptide residual Z score: 0.154
Number of peptides: 5841
Pred. hydrophobicity
60
40
20
0
0
25000
50000
75000
Experimental scan number
C
Distributions of residuals from best−fit line
of predicted RT vs Expt. scan number
Line: Z score of novel peptide
Z: 0.154
count
1500
1000
500
0
−10
−5
0
5
10
Z scores of residuals from best linear fit
